# Modeling of Effect of Infill Density Percentage on Rotating Bending Fatigue Behavior of Additive-Manufactured PLA Polymers

**DOI:** 10.3390/ma17020471

**Published:** 2024-01-19

**Authors:** Ezzeddine Ftoutou, Lamis Allegue, Haykel Marouani, Tarek Hassine, Yasser Fouad, Hatem Mrad

**Affiliations:** 1 Mechanical Engineering Laboratory, National Engineering School of Monastir, University of Monastir, Monastir 5019, Tunisia; ezzeddine.ftoutou@enim.rnu.tn (E.F.); lamiss.allegue@enim.u-monastir.tn (L.A.); tarek.hassine@enim.u-monastir.tn (T.H.); 2 Department of Applied Mechanical Engineering, College of Applied Engineering, Muzahimiyah Branch, King Saud University, P.O. Box 800, Riyadh 11421, Saudi Arabia; yfouad@ksu.edu.sa; 3 School of Engineering, University of Québec in Abitibi-Témiscamingue (UQAT), Rouyn-Noranda, QC J9X 5E4, Canada; hatem.mrad@uqat.ca

**Keywords:** fatigue properties, PLA polymer, 3D printing, Wöhler model, Basquin model

## Abstract

Nowadays, 3D PLA-printed parts are widely used in many applications, essentially using the fused filament fabrication technique. While the influence of printing parameters on quasi-static mechanical characterization has been extensively considered within the literature, there are limited accounts of this effect on fatigue performance. The two main aims of this research are first to investigate the effects of the infill density percentage on the fatigue life of dog-bone samples under rotating bending cycling loads, and second to model the fatigue life using Wöhler and Basquin models. The experiments exhibit a high variability of results, especially for low cyclic loads. The S–N curves show that the number of cycles at failure increases with the increase in the infill density percentage and decreases with the increase in loads. Investigations allow the formulation of each constant model as a function of the infill density percentage. The new fatigue model formulations exhibit good agreement with the experimental data. As an outcome of this study, the fatigue model for 3D-printed parts may be expressed as a function of the infill density percentage using fewer tests in the future and for other polymers used in fused filament fabrication.

## 1. Introduction

The use of additive manufacturing (AM), commonly named 3D printing, is becoming more widespread in several industries with severe production and standards constraints, namely aerospace [1,2], automotive [3,4], biomedical [5,6], healthcare and medical devices [7,8], architecture and construction [9,10], electronics [11,12], and many others. For example, according to General Electric Aerospace, the number of parts produced using traditional manufacturing methods was cut from 855 to 12 using AM technologies. AM simplified the design, reduced the weight, improved fuel efficiency by up to 20%, and achieved 10% more power [13]. Similar to that, Airbus reduced a hydraulic housing tank that had 126 parts to having just 1 AM part [1]. Flapping wings, satellite brackets and lightweight components for the aviation industry may now be produced easily thanks to advancements in composite and multi-material production [2]. Fiber-reinforced AM is nowadays used to generatively develop and produce a race car’s highly stressed and safety-relevant chassis part [14]. In the biomedical field, 3D printing has been widely used to create customized prosthetics [15], dental implants [16], and organ and tissue fabrications [17].

In fact, there are many types of AM techniques. Each has its own advantages and limitations, making them suitable for different applications within various industries [18,19,20]. Among them, we cite stereolithography, selective laser sintering, binder jetting, direct metal laser sintering, electron beam melting, powder bed fusion, continuous liquid interface production, and fused filament fabrication (FFF). FFF, also known as fused deposition modeling (FDM) or material extrusion (MEX), is one of the most popular and economical ways to manufacture plastic parts among the different AM processes; a continuous thermoplastic or composite material thread in a filament form is used to manufacture 3D items [21,22]. This process uses an extruder to feed polymer filament via an extruding nozzle, which melts it and then deposits it in a specified automated path, layer by layer, onto the build platform. 

Many commercial polymers are used in FFF, such as acrylonitrile butadiene styrene (ABS), polylactic acid (PLA), polycarbonate (PC), polyether ester ketone, polyetherimide, acrylonitrile styrene acrylate, polyethylene terephthalate glycol, and thermoplastic elastomers [23,24,25,26]. However, due to its simplicity of use and low expansion and contraction upon heating and cooling, PLA is the most often used material. Indeed, it has a relatively low printing temperature, typically around 180–220 °C, which makes it compatible with a wide range of 3D printers, including those with open-frame designs. Its low printing temperature also means that it has less tendency to warp during printing. PLA is also biodegradable, with low odor and low toxicity when heated. Moreover, it is available in a wide range of colors and compatible with dual extruders (when combined with a water-soluble support material like polyvinyl alcohol [27,28]). It is important to note that while PLA has many advantages, it also has limitations. For example, its low heat resistance, compared to some other 3D printing materials like ABS or PC, makes it not suitable for applications exposed to high temperatures. Its low flexibility and toughness also limit its application [29].

Due to the spread of 3D-printed parts, their mechanical characterization is essential for material selection, product design, quality control, performance evaluation, and research. They play a central role in ensuring the safety, reliability, and efficiency of materials and structures across various industries. Tensile, compression, flexural, shear, hardness, and fatigue tests are examples of the many mechanical characterization tests available. As one of the most frequent reasons for structural and mechanical components prematurely failing is the phenomenon of fatigue. In fact, fatigue tests remain among the most interesting. Fatigue material characterization, proposed initially by Whöler, consists of experimental campaigns based on standardized specimens suggested by international standards subjected to dynamic loading until failure. A critical parameter related to dynamic loading is defined, such as stress, σ; stress range, Δσ; or strain range, Δε. Then, a relation between the critical parameter and the number of cycles until failure is defined by a fatigue life model. The fatigue models can be formulated in terms of σ–N, called S–N curves. The proposed models in the literature can be classified into two groups [30]. The first group belongs to the deterministic models, such as the Basquin, Palmgren, Stromeyer, and Weibull models. The second group belongs to the probabilistic models, like the Bastenaire, Castillo and Canteli, Bolotin, and Pascual and Meeker models. 

According to [23], the PLA tensile strength is around 55 MPa, the modulus of elasticity ranges from 827 to 1552 MPa, the elongation to failure ranges from 5.1 to 16.6%, the shore D hardness ranges from 65.8 to 81.7 HShD, and the ball indentation hardness varies between 77.2 and 99.6 N·mm^−2^. This variability in results is due to the PLA supplier and the applied 3D printing parameters. Indeed, the FFF printing process depends on 13 parameters that affect the mechanical performance of the product. These parameters can be categorized into two classes [31]: the layering and device parameters. The first class refers to the topological parameters that can be controlled by the slicing software, such as the raster angle, the print direction (also called build orientation), the infill pattern, the infill density, the layer height, the extrusion width, the air gap, the solid layers, and the perimeters. The second class refers to the parameters associated with the 3D printing device itself, such as the print speed, the nozzle diameter, the nozzle temperature, and the platform temperature. While the influence of FFF printing parameters on quasi-static mechanical characterization has been widely discussed within the literature [31,32,33], there are limited accounts of this effect on fatigue performance. For example, Azadi et al. [34] investigated the impact of the print direction on the bending fatigue properties of PLA: a shorter fatigue lifetime was obtained for vertical specimens than that of horizontal samples. Shanmugam et al. [35] investigated the effect of the nozzle diameter, the extrusion temperature, the bed temperature, the extrusion speed rate, and the layer height on fatigue. They concluded that the printing parameters have a significant impact on fatigue behavior, which need to be optimized. They also concluded that the defects and voids are common problems in FFF, and would be considered factors since they increased the stress concentration. 

Infill density plays a crucial role in determining the mechanical properties of 3D-printed parts. Indeed, increasing infill density generally improves the overall strength and stiffness of the printed part. The reason is that a higher infill creates a more solid interior structure, reducing the likelihood of deformation and providing better support for the outer layers. However, it also increases the weight and material usage of the printed part. This can affect the cost, print time, and overall efficiency of the 3D printing process [36]. Infill patterns (square, hexagonal, triangular, etc.) also significantly influence the mechanical properties of printed parts [37]. Gomez-Gras et al. [38] investigated the fatigue performance of PLA cylindrical specimens using Taguchi Design of Experiments. They concluded that infill density emerges as the predominant factor affecting fatigue performance, followed by nozzle diameter and layer height. Printing speed has a negligible influence on PLA specimens.

In this paper, an experimental campaign is realized to characterize the fatigue behavior under rotating bending tests for PLA samples obtained via FFF. Four levels of the infill density percentage (f_%_) of dog bone samples (25%, 50%, 75%, and 100%) are tested. Wöhler and Basquin models are used to express S–N data. Updated expressions of the cited models are implemented to consider the effect of infill density on fatigue model constants. The new formulations are validated with success according to extra experimental configuration tests.

## 2. Experimental Set-Up and Analysis Methodology 

This section describes the geometrical and material properties of the 3D-printed samples, the rotating bending set-up, and the S–N models used in this study.

### 2.1. Fatigue Samples

**Figure 1:**
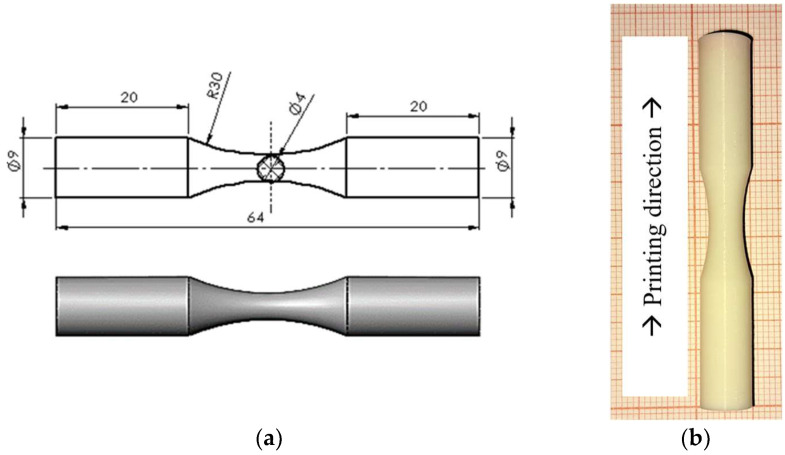
(**a**) Fatigue dog bone sample; (**b**) PLA specimen printed in vertical direction.

Fatigue samples are FFF-manufactured using a Rise3D Pro2 printer. The study material is commercial PLA (Anet, Shenzhen, China), which is injected layer by layer. Vertical divisions of the normal dog bone samples are applied. Figure 1 illustrates the dog bone sample dimensions (based on the ISO 1143:2010 standard [39]) and the manufactured specimen.

The 3D printing parameters are settled in accordance with the literature and the PLA supplier recommendations [34,40]. The nozzle temperature is fixed at 200 °C and the bed temperature at 60 °C. The nozzle speed is 50 mm/s, and the nozzle diameter outlet is equal to 0.4 mm. A square pattern fills the material with a layer thickness of 0.15 mm. The infill density of the samples is 100% in the initial and final layers. The inner layers are filled to 25, 50, 75, and 100% density. The filling speed is equal to 60 mm/s. The raster orientation is −45/45°.

### 2.2. Fatigue Testing

**Figure 2:**
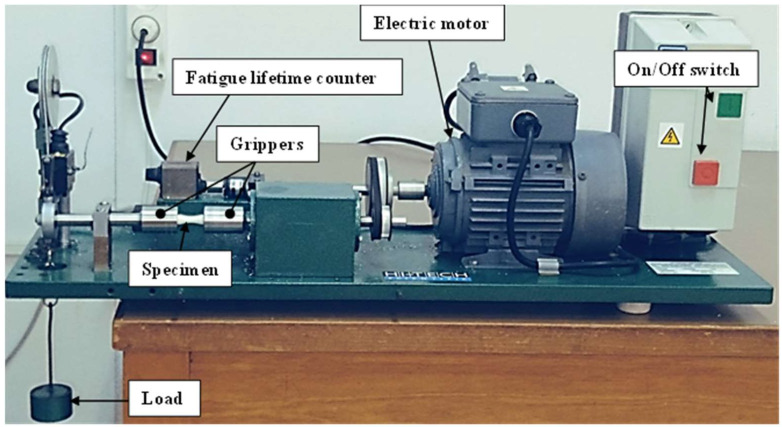
Rotating bending fatigue device.

A rotating bending fatigue device is used to establish the S–N diagram. The apparatus (Company: HI-TECH LIMITED, Andover, UK, model: HS M-19, Figure 2) contains a cantilever beam condition in which the bending stress is applied to the shaft end as a load. The maximal bending stress on the specimen surface is totally reversed since the sample is rotating (R = −1, which means the mean stress is zero). The bending load is set at 2, 5, 7 and 10 N, corresponding to an alternate peak bending stress of 14, 35, 49 and 70 MPa. The loading frequency is set to 100 Hz. Each experiment is conducted 10 times to investigate the repeatability of the fatigue results. In total, 160 fatigue tests are carried out for this study.

### 2.3. Fatigue Life Modeling

There are numerous methods and models with which to estimate the fatigue life. This section presents two deterministic fatigue life prediction models that express the Wöhler S–N curve. The first one, called the Wöhler model [41], was established in 1870 and it represents the S–N data as follows:

(1) $$Log\left(N_{f}\right)=C_{W1}-C_{W2}\sigma$$

where σ is the stress amplitude, *N_f_* is the number of cycles to failure, and $C_{W1}$ and $C_{W2}$ are Wöhler material positive constants.

The second model is called the Basquin model [42]. It was established in 1910 and it expresses the S–N data in the following equation:

(2) $$Log\left(N_{f}\right)=C_{B1}-C_{B2}Log(\sigma )$$

where $C_{B1}$ and $C_{B2}$ are Basquin material positive constants.

## 3. Results and Discussion

In this section, we discuss the size of the fatigue samples used to characterize the S–N curves accurately. Then, we present our investigation on fatigue behavior modeling. Two models, Wöhler and Basquin, are used. The material constants are expressed as a function of the infill density percentage (f_%_) to formulate a unique equation that considers its contribution to the model formulation. The results are validated with additional experiments.

### 3.1. Fatigue Specimen Sampling

**Figure 3:**
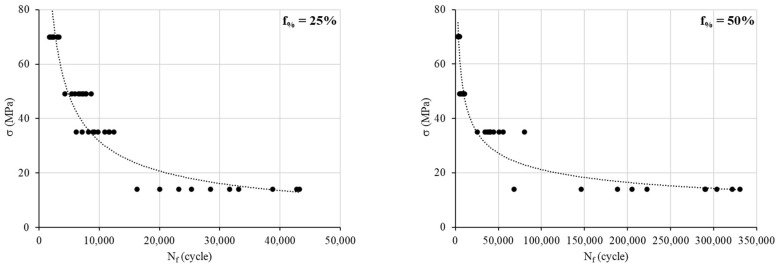

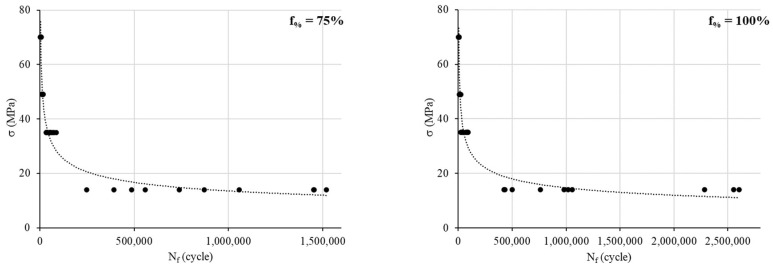
S–N curves for different infill density percentages.

Fatigue tests show an inevitable variability in the number of cycles at failure, especially for the low stress of 14 MPa (see Table 1 and Figure 3). Yet, the number of samples that must be tested depends on three parameters: the variability of observed results, the desired accuracy, and the desired level of confidence for the estimated result. Very often, the desired accuracy is expressed as a percentage of the mean of the observed result. For example, the ambition of fatigue modeling may be to achieve an estimate within 10 percent of the actual mean. The sample size needed to achieve that purpose can be determined using the following formula:

(3) $$n={\left(\frac{z\cdot s}{a\cdot \bar{N_{f}}}\right)}^{2}$$

where *z* is the number of normal standard deviations needed for desired confidence, *s* is the sample standard deviation, *a* is the desired accuracy percentage, and $\bar{N_{f}}$ is the mean of the number of cycles at failure.

Primarily, 10 tests are performed for each configuration to evaluate the standard deviation and then to update the correct required number of samples regarding the desired confidence targeted. Table 1 resumes the number of cycles at failure under the peak stress of 14 MPa, for instance, and the corresponding sample size. We opt for a desired confidence of 90% (z = 1.65) and a desired accuracy percentage of ±10%. For the infill density percentage of 75% and 100%, the required number of samples are 79 and 132, respectively. This means that we should make 69 and 122 more fatigue tests for each of these configurations. Unfortunately, these tests are time-consuming, so we decided to limit our investigations to the initial 10 tests already made.

### 3.2. Fatigue Behavior Modeling

Based on the rotating bending tests, the S–N curves are obtained (Figure 3). The data show a significant variability in the *N_f_* cycles for a low load (14 MPa). Indeed, the ratio of *N_f min_* to *N_f max_* at 14 MPa is 2.66, 4.84, 6.10 and 6.13 for infill percentages of 25, 50, 75 and 100%, respectively. However, for the other loads, this ratio ranges between 2 and 3.8, between 1.5 and 2.2 and between 1.5 and 2.2 for 35 MPa, 49 MPa and 70 MPa, respectively.

**Figure 4:**
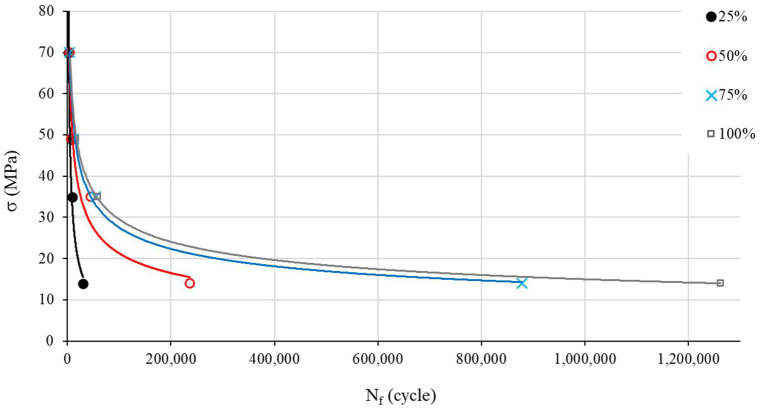
S–N curves for different infill density percentages (mean values).

**Figure 5:**
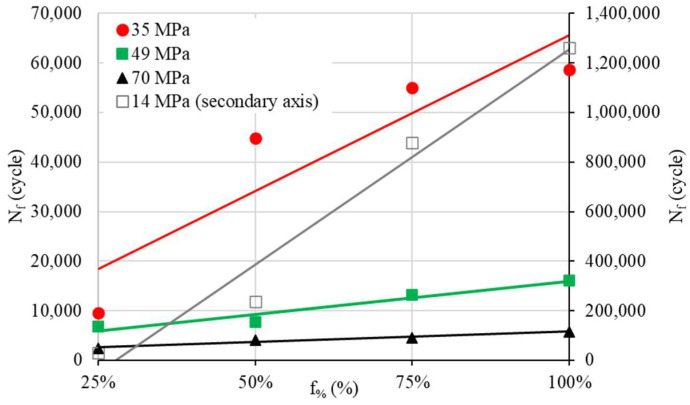
*N_f_* evolution as a function of f_%_ for different cyclic loads.

Our following investigations are based on the mean values of *N_f_* (Figure 4). The S–N curves show the expected material behavior under a cyclic load: *N_f_* logically goes up with the increase in f_%_ and decreases with the rise in the load (see Figure 5).

#### 3.2.1. Wöhler Model

**Figure 6:**
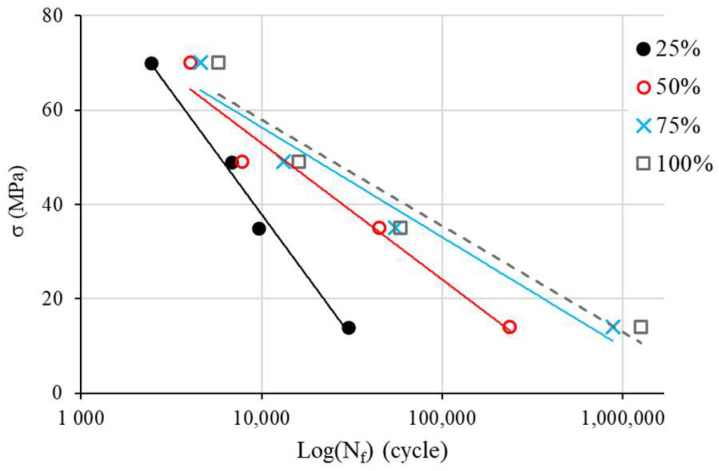
S-log(N) curves for Wöhler model identification.

To assess Wöhler coefficients, we start by depicting the S–N curve with a Log(N) scale (Figure 6) in accordance with Equation (1). The relationship is obviously linear. The use of the linear regression allows the determination of $C_{W1}$ and $C_{W2}$ (Table 2, R^2^: coefficient of determination).

**Figure 7:**
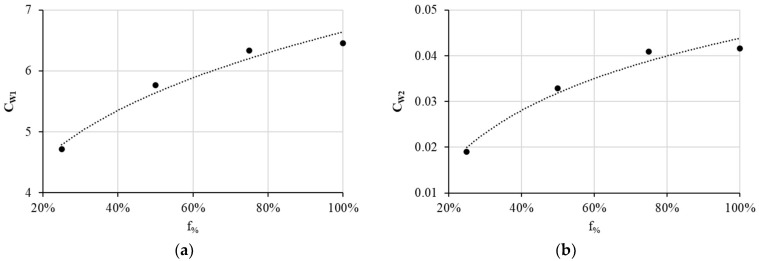
Evolution as a function of f_%_ of (**a**) $C_{W1}$ and (**b**) $C_{W2}$.

Figure 7 depicts $C_{W1}$ and $C_{W2}$ as a function of f_%_. Equations (4) and (5) express the obtained least squares regression with a coefficient of determination of 0.96 for $C_{W1}$ and of 0.97 for $C_{W2}$.

Thus, we can express Wöhler material positive constants as follows:

(4) $$C_{W1}\left(f_{\%}\right)=6.64f_{\%}^{0.235}$$

(5) $$C_{W2}\left(f_{\%}\right)=0.0438+0.017Ln(f_{\%})$$

#### 3.2.2. Basquin Model

**Figure 8:**
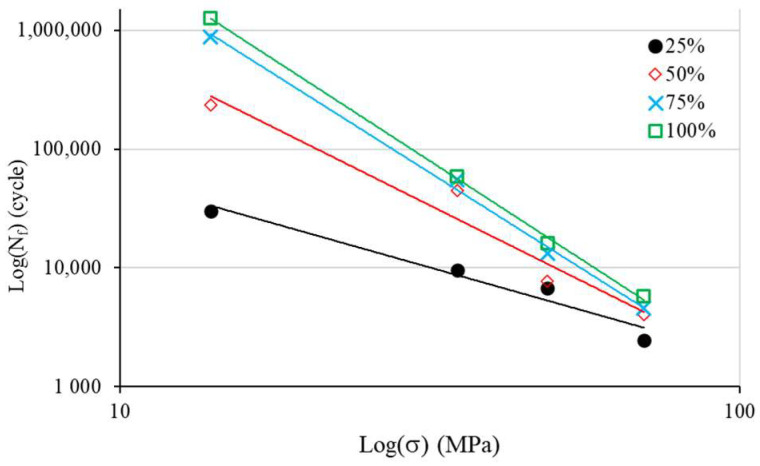
Log(S)–log(N) curves for Basquin model identification.

To assess Basquin coefficients, we now depict the S–N curve with a log(S)–log(N) scale (Figure 8) in accordance with Equation (2). The relationship is obviously linear. The use of linear regression allows the determination of $C_{W1}$ and $C_{W2}$ (Table 3).

**Figure 9:**
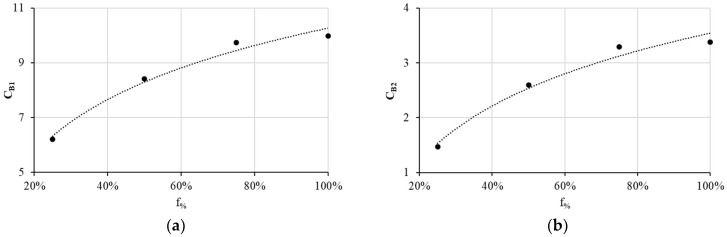
Evolution as a function of f_%_ of (**a**) $C_{B1}$ and (**b**) $C_{B2}$.

Figure 9 depicts $C_{B1}$ and $C_{B2}$ as a function of f_%_. Equations (6) and (7) express the obtained least squares regression with a coefficient of determination, R^2^, of 0.98 for $C_{B1}$ and of 0.97 for $C_{B2}$.

Hence, we can express the Basquin material positive constants as follows:

(6) $$C_{B1}\left(f_{\%}\right)=10.266+2.841Ln(f_{\%})$$

(7) $$C_{B2}\left(f_{\%}\right)=3.543+1.451Ln(f_{\%})$$

#### 3.2.3. Validation of Models

**Figure 10:**
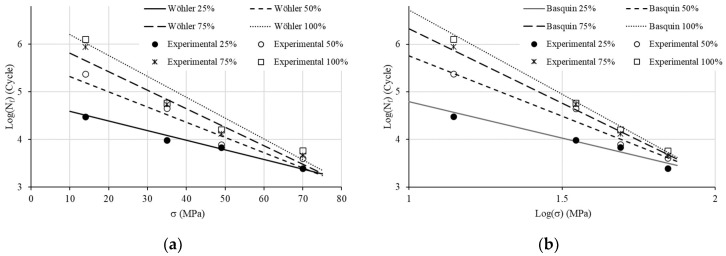
Theorical formulation and experimental data: (**a**) Wöhler and (**b**) Basquin.

Combining Equations (1), (4) and (5), and combining Equations (2), (6) and (7) allow us to formulate Wöhler and Basquin models, respectively. Figure 10 shows good agreement between the theoretical models and experiments. To validate the new formulations, an additional experimental campaign is performed: two stress levels are used (28 and 63 MPA) at different infill density percentages ranging from 35% to 85% (Table 4). Each test is repeated only five times.

**Figure 11:**
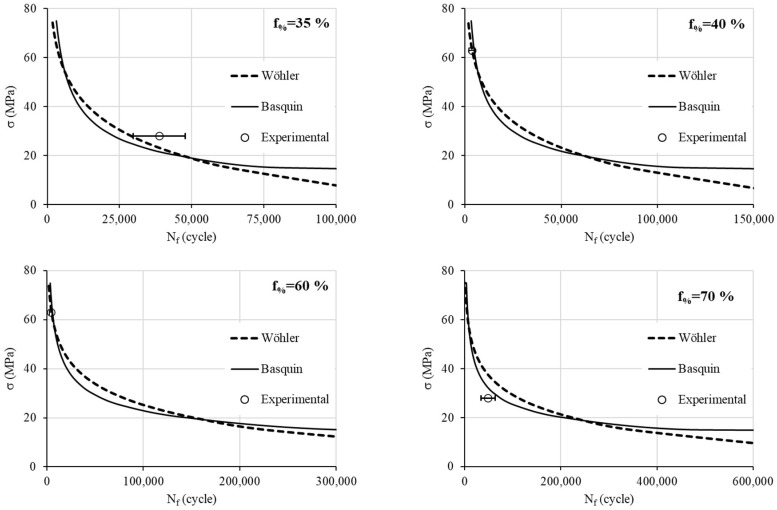

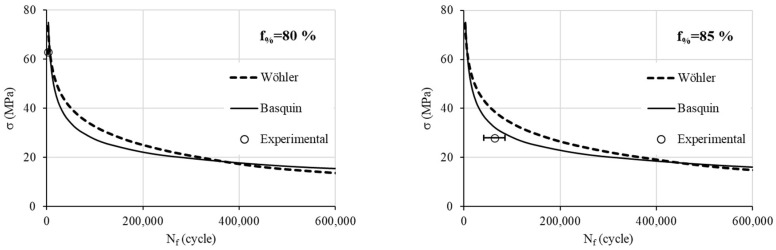
Theorical formulation at different infill density percentages and experimental data.

Figure 11 represents the fatigue models with the experimental values. The experiments are depicted by their mean value and the error bars (standard deviation). We note that for the high stress of 63 MPa (a low number of loading cycles), both theoretical models perfectly represent the fatigue behavior. However, for the low stress of 28 MPa, we note that for the density infill percentage of 35%, Wöhler shows a better agreement than Basquin does. For higher density infill percentages, Basquin becomes more realistic. We recall that for the low stress, three replications are insufficient to model the high variability of observed results.

The major cause of the deviations between the model and actual experimental results is attributed to the discrepancies in the experimental data themselves. Specifically, the fatigue tests exhibit a high degree of deviation, especially at a high number of cycles. Additionally, the deviation can be ascribed to the properties of the material used. Indeed, the mechanical properties of PLA, like all polymers, are temperature-dependent, and any change in room temperature can impact the overall fatigue results.

## 4. Conclusions

FFF is essential due to its versatility and wide-ranging applications in various industries. One of the most used materials is PLA due to its ease of use, eco-friendliness, good surface finish, and affordability. PLA 3D prints must be assessed regarding mechanical behavior, essentially under repeated cyclic loading and unloading. However, the overall ability to withstand repeated stress or load cycles over time depends on the FFF parameters. In this work, we focus on how the infill percentage density affects the fatigue life of dog bone samples under rotating bending tests. The experimental data show a high variability of results, which requires a significant number of replications for each test to improve the confidence and accuracy of outputs. In some cases, hundreds of tests are required for confidence and accuracy with 90% for each. However, due to time constraints, we limit our experimental campaign to 10 replications per configuration. The S–N curves show that the number of cycles upon failure goes up with the increase in the infill density percentage and decreases with the rise in the load. Two deterministic fatigue life prediction models are used: Wöhler and Basquin. The investigations demonstrate that a unique formulation of the cited models can be expressed to consider the effect of the infill density percentage. Indeed, the model constants can be related to it. The results show that both models agree on high cyclic loads, which correspond to the low-cycle fatigue regime. However, for the low cyclic load, which corresponds to the high-cycle fatigue regime, the new Wöhler formulation shows a better fit for an infill density percentage lower than 50%. For the other cases, higher than 50%, the new Basquin formulation is better. This finding means that a fewer number of tests can be performed to express the fatigue model as a function of the infill density percentage. According to the literature, decreasing surface roughness using a post-processing approach (by turning) would enhance the 3D print lifetime through the high-cycle fatigue regime. Future research will analyze the influence of 3D-printed surface roughness samples on the fatigue life.

## Figures and Tables

**Table 1:** Size sample (desired confidence of 90% and desired accuracy percentage of ±10%).

	Infill Percentage			
Specimen N°	25%	50%	75%	100%
1	25,300	146,050	249,250	1,020,750
2	43,200	289,800	1,454,800	424,750
3	20,000	222,400	485,300	982,700
4	23,200	68,200	1,451,940	762,700
5	42,800	321,600	392,150	1,055,780
6	16,250	205,100	1,057,400	2,284,000
7	28,500	330,300	1,520,200	2,605,000
8	31,600	188,100	872,300	2,552,300
9	38,800	290,600	558,350	435,000
10	33,100	303,700	740,150	499,150
mean	30,275	236,585	878,184	1,262,213
standard deviation	9349	85,866	472,994	876,096
n	26	36	79	132

**Table 2:** Identification of Wöhler coefficient for different infill percentages.

	$C_{W1}$	$C_{W2}$	R^2^
25%	4.719	0.0190	0.987
50%	5.763	0.0329	0.950
75%	6.336	0.0409	0.952
100%	6.460	0.0417	0.936

**Table 3:** Identification of Basquin coefficients for different infill percentages.

	$C_{B1}$	$C_{B2}$	R^2^
25%	6.206	1.4678	0.955
50%	8.420	2.5953	0.956
75%	9.740	3.2922	0.996
100%	9.974	3.3825	0.999

**Table 4:** New configurations for validation.

σ (MPa)	$f_{\%}$ (%)
28	35
28	70
28	85
63	40
63	60
63	80

## Data Availability

Data are contained within the article.
